# Medical and Philosophical Intelligence

**Published:** 1815-09

**Authors:** 


### MEDICAL AND PHILOSOPHICAL INTELLIGENCE.

### APOTHECARIES' HALL.

*Regulations for the Examination of Apothecaries.*

THE Court of Examiners, chosen and appointed by the Master, Wardens, and Assistants, of the Society of Apothecaries, of the City of London, in pursuance of a certain Act of Parliament, "For better Regulating the Practice of Apothecaries throughout England and Wales," passed in the 55th year of the reign of his Majesty King George the Third, has determined—

That every person who shall be admitted to an examination for a certificate to practice as an apothecary, shall be required to produce testimonials of having served an apprenticeship of not less than five years to an apothecary; of having attained the full age of twenty-one years; and being of a good moral conduct. He is expected to possess a competent knowledge of the Latin language, and to produce certificates of having attended not less than two courses of Lectures on Anatomy and Physiology; two courses of Lectures on the Theory and Practice of Medicine; one course of Lectures on Chemistry; and one Course of Lectures on Materia Medica. A certificate of attendance, for six months at least, on the medical practice of some public Hospital, Infirmary, or Dispensary.

The Court has also determined, that the examination for a certificate to practice as an apothecary shall be as follows:

1. In translating parts of the Pharmacopœia Londinensis, and Physicians' Prescriptions.
2. In the Theory and Practice of Medicine.
3. In Pharmaceutical Chemistry.
4. In the Materia Medica.

*Regulations for the Examination of Assistants.*—That every person who shall be admitted to an examination for a certificate to act as an assistant to any apothecary, in compounding or dispensing medicines, shall be required to translate parts of the Pharmacopœia Londinensis and Physicians' Prescriptions; and shall be examined as to his knowledge of Pharmacy and Materia Medica.

*Notice.*—Every person intending to qualify himself under the regulations of this Act to practice as an apothecary, or to act as an assistant, must give notice in writing (post paid), addressed to the the Clerk of the Society of Apothecaries, Apothecaries' Hall, London, at least six days previously to the day of examination.

The Court will meet in the Hall on Thursday the 3d of August, at two o'clock of the afternoon precisely, and on every following Thursday at the same hour. By order of the Court,

*London, July* 31, 1815. JOHN WATSON, Secretary.

It is expressly ordered by the Court of Examiners, that no gratuity be received by any officer from any person applying for information relative to the business of this Court.

EXTRACTS *from the Act* " *For better Regulating the Practice of Apothecaries throughout England and Wales.*"

" That, from and after the first day of August, one thousand eight hundred and fifteen, it shall not be lawful for any person or persons (except persons already in practice as such), to practise as an apothecary in any part of England or Wales, unless he or they shall have been examined by the Court of Examiners, or the major part of them, and have received a certificate of his or their being duly qualified to practise as such, from the said Court of Examiners; who are authorized and required to examine all person or persons applying to them, for the purpose of ascertaining the skill and abilities of such person or persons in the science and practice of medicine, and his or their fitness and qualification to practise as an apothecary.

" That from and after the first day of August, one thousand eight hundred and fifteen, it shall not be lawful for any person or persons (except the persons then acting as assistants to any apothecaries, and excepting persons who have actually served an apprenticeship of five years to an apothecary) to act as an assistant to any apothecary, in compounding or dispensing medicines, without undergoing an examination by the Court of Examiners, or by five apothecaries, so to be appointed as hereinafter is mentioned.

" That it shall and may be lawful to appoint five apothecaries in any county or counties respectively throughout England and Wales (except within the said city of London, the liberties or suburbs thereof, or within thirty miles of the same), to act for such county or counties, or any other county or counties near or adjoining; and such five apothecaries are authorized and empowered to examine all assistants to apothecaries throughout the county or counties in regard of which such apothecaries shall have been so appointed as aforesaid.

" That if any person (except such as are then actually practising as such) shall, after the said first day of August, one thousand eight hundred and fifteen, act or practise as an apothecary in any part of England or Wales, without having obtained such certificate as aforesaid, every person so offending shall, for every such offence, forfeit and pay the sum of twenty pounds; and if any person (except such as are then acting as such, and excepting persons who have actually served an apprenticeship as aforesaid) shall, after the first day of August, one thousand eight hundred and and fifteen, act as an assistant to any apothecary, to compound and dispense medicines, without having obtained such certificate, every person so offending, shall for every such offence forfeit and pay the sum of five pounds.

"That no apothecary shall be allowed to recover any charges claimed by him in any court of law, unless such apothecary shall prove on the trial, that he was in practice as an apothecary prior to, or on the said first day of August, one thousand eight hundred and fifteen, or that he has obtained a certificate to practise as an apothecary.

"That the said Master, Wardens, and Society of Apothecaries, do make annually, and cause to be printed, an exact list of all and every person who shall in that year have obtained a certificate to practise as an apothecary, with their respective residences attached to their respective names."

N.B. It may not occur to every reader that the above notice has no retrospect to persons already engaged in practice, or as assistant apothecary.

We have received from "*An Apprentice*," and a great number of other correspondents, remarks on the Apothecaries' Bill. Like most other Acts, it is, probably, liable to some objections. As the Act itself is too full of technical verbiage for the room we could spare, and not without the attendant obscurity of other Acts, it is our intention to procure a perspicuous abstract of it, which will render any remarks intelligible. We shall, therefore, thankfully receive every remark, and insert them verbatim, if short, and the more pointed parts, if long.

---

Mr. WACHSELL's letter having arrived too late for insertion in our last, it is now only necessary to insert the following advertisement, which has appeared in several of the daily papers.

*Hospitals for the Small-pox, for Inoculation, and for Vaccination, St. Pancras.*—At a Committee held at this Hospital, on Thursday, the 3d day of August, 1815, a Report, dated 19th June last, from the National Vaccine Establishment, was laid upon the table, at the foot of the third page of which is contained the following note:—"The Small-pox Hospital has been lately purchased for the use of the sick poor, afflicted with fevers."

The Committee, being fearful lest the above note should excite an erroneous opinion that this Hospital is no longer open for the reception of the poor as usual, think it right to acquaint the public, that since the increased confidence in vaccination, the numbers vaccinated at this Hospital have greatly increased, and those inoculated have diminished in the same proportion; and hence the additional wing built since this original spacious establishment, being found unnecessary, is disposed of to the Fever Institution.

That the original building is larger than necessary for all the departments of this institution; and that, should an epidemic occur, equal to any for the last ten years, it will be sufficient for the accommodation commodation of double the number of patients who have hitherto applied for relief.

Patients are admitted, and information given, as usual, at this Hospital daily. By order of the Committee.

A. Highmore, Secretary.

---

*To the Editor of the Medical and Physical Journal.*

SIR,

By publishing the following simple method of preserving, or rather of recovering, cheese, after it has been rendered totally useless by the effects of a hot climate, (which is very soon the case,) you would no doubt oblige several of your seafaring readers, as you would both enable them to enjoy an agreeable article of food, when they could not otherwise have it; and also prevent much of it from being totally lost, which is but too often the case, as I have more than once had occasion to witness.

Steep the cheese for ten or twelve days in the pickle of salt beef, after the meat is all taken out; then hang it up in a bread-bag, out of the sun, and, if possible, in a current of air, till it is well dried, when it will be found perfectly firm, pleasant to the taste, and not much salter than before the operation,—although it had been quite fetid, moist, and so soft that the fingers might have easily been pushed through it.

I am unable to say how long it might continue good afterwards, or whether a repetition of the process would recover it a second time, as ours continued perfectly sound as long as there was any of it remaining, though in the West Indies, during the summer season.

J. K., R. N.

---

*Account of a Polypus of the Stomach which was rejected by Vomiting;* by M. Fabre *—A lady of Provence, thirty years of age, was attacked, about twelve years ago, with pains and cramps in the stomach, which always became more acute and lancinating two or three days before the periods of menstruation. At these times she could swallow fluids only, the pains of the stomach being much increased by swallowing solids, which were almost immediately rejected by the most violent strainings. The flow of the courses had been nevertheless remarkably easy.

At no time could she drink spirituous liquors, or even wine, without experiencing an almost insupportable burning heat at the epigastrium.

The physicians who had been consulted on the nature of the case, were satisfied that the symptoms indicated a chronic inflammation of the stomach, *(un etat permanent de phlogose du ventricle,)* the cause of which was not evident, although they conceived there might be a dilated state of the blood-vessels of that viscus, and consequently an inordinate excitement of the nervous system.

---

* Gazette de Santé.

Under

Under this belief, they prescribed copious bleedings, acidulated and lightly narcotic fluids, the application of opium plasters and compresses with æther to the pit of the stomach, baths, purgative glysters, blisters upon the arms and thighs, and various other remedies. Notwithstanding, however, the employment of these means, the pains of the stomach increased to such an alarming degree, that, for the last eighteen months, the patient was supported entirely on animal jellies, panada, and broth glysters. When I first saw her, which was on the 8th of January last, at seven o'clock in the morning, she was extremely emaciated and weak.

Nevertheless, she was in the fifth month of her pregnancy; and symptoms of abortion had come on, attended with vomiting.

I was extremely uneasy at finding I was the only practitioner in attendance on this unfortunate woman; and felt a conviction that I should witness her death perhaps even before the fœtus was expelled. I therefore requested a consultation; but both the patient and her mother opposed my wishes, and I was obliged to yield to their entreaties.

I had been three hours with my patient, when she complained of an intolerable tearing pain of the stomach. The nausea and vomitings became more and more frequent, their intensity apparently corresponding with that of the uterine action.

Soon afterwards, she vomited a great quantity of blood; and after the severest strainings, threw up a fleshy substance, of the size of a hen's egg, and somewhat resembling a cauliflower, with a very slender peduncle, by which it most probably adhered to the stomach. The base of the stem was the only part of the tumour which was in any degree lacerated.

The abortion terminated almost immediately after the expulsion of the tumour.

During the remaining part of the day, she had slight nausea, accompanied with a sensation of irritation or of acute pain at the stomach.

This irritation gradually subsided, and was succeeded by a purging, which I allowed to take its course; and which continued until the eleventh day.

The fever, which generally occurs on the third day after delivery, scarcely shewed itself; there was no indication of the secretion of milk, and the lochial discharge was as usual.

On the twentieth day, the patient ate some soup, bread, and meat; and did not afterwards feel any fatigue or sensation of weight at the stomach, of which she had complained until the fourteenth day.

On the thirtieth, she felt no kind of pain whatsoever, and since that time, after having tried gradually the powers of the stomach, she has indulged her appetite, and eaten of all kinds of dishes. She becomes fat, and begins to enjoy good health.

---

The Gazette de Santé, dated the 21st of June, contains two cases of laryngitis, *angine laryngée œdemateuse*, *Gonflement ou œdeme de*

*de la glotte*, which occurred in the Hotel Dieu, during a cold, rainy, and very variable season. A young man, convalescent from fever, early in the morning took cold; at noon he complained of general uneasiness, slight chills, and heat in the bottom of the throat; at four o'clock felt acute pain in the larynx; inspiration was performed with great pain, but no difficulty was felt in expiration; the voice was shrill and tremulous, deglutition difficult, countenance florid, pulse contracted and frequent. Five leeches were applied to the neck, and afterwards a cataplasm. He was not relieved by these means, had no sleep, and his anxiety was very great. On the following day his symptoms had increased; countenance was of a livid hue; expiration tolerably easy. The patient held his head backward, to facilitate inspiration, which was performed with inexpressible anguish; the pulse was hard, small, and frequent. Five leeches and a blister were applied. He died at two o'clock. After death, the face, neck, and head, were suffused with blood. On dissection, the mucous membrane of the epiglottis, glottis, and ventricles of the larynx, were inflamed and thickened. The cellular texture anterior to and at the root of the epiglottis was so large, that the latter was pushed back toward the opening of the larynx, which was much contracted; the trachea was healthy. If we had not such frequent specimens of the want of energy in the practice of the French physicians, we should suspect that man of idiotcy who could suffer inflammation of the larynx, to run its course without the employment of blood-letting. If any disease require depletion to its fullest extent, it is this in its acute state, which makes such rapid strides towards its fatal termination. The unutterable anguish, suffusion of the countenance, and gasping for breath, ought to be sufficient to induce at least a trial of the remedy, even if the inflammatory character of the disease were not so distinctly marked. We observe in the same narrative, a striking confirmation of the inconsistency of the attending physician. A case of angina trachealis (croup) in an adult, is adduced for the purpose of instituting a comparison between the two affections so nearly resembling each other in name. This case, compared with the former, is not remarkable for intensity of suffering, or great danger; yet, on the fourth day of the disease, fifteen leeches, and, on the fifth, twenty more were applied to the throat, with advantage. Now, if thirty-five leeches are necessary for the cure of a case of angina trachealis, we are at a loss to conceive upon what principles of calculation our continental friend discovers ten to be sufficient for the cure of that horrid assemblage of symptoms characteristic of angina laryngea. The reporter very properly recommends bronchotomy in the latter, as the means of averting suffocation. It will be obvious, that if the larynx be obstructed so as to make the ingress of air into the lungs difficult or impossible, this operation is our only resource, our past experience does not indeed warrant an expectation of cure from it; but, so long as the opening in the trachea is preserved, the symptoms are very considerably abated, as might, *à priori*, be expected.

expected. We submit to the consideration of our brethren in the profession, whether it should not be performed before the disease had arrived at its acme; the suffering of the patient would in a great measure be removed *in limine,* and perhaps a source of irritation destroyed, which may have a tendency to aggravate this disease.—*New England Journal.*

---

The following is said to be a very efficacious application for rheumatism, and is known by the name of Sanchez's Balsam:—Aromatic soap, an ounce; spirit of lavender, four ounces; camphor, two ounces; essential oils of peppermint, canelle, lavender, muscade, quassia, sassafras, of each fifteen drops; acetic æther, an ounce. The editor of the *Journale Generale de Medecine,* from whom we take the account, considers the efficacy of the remedy to reside principally in the acetic æther.

---

The Hepar sulphuris has been successfully applied by M. Bertrand to tettery eruptions *(affections dartreuses).* As the French term *dartre* implies a variety of affections, we subjoin the description of two cases in which it was useful.

1st. *Dartre rongeante.*—A woman of fifty-seven had for seven years past suffered from a continual itching in the external parts of generation. On examination, little ulcerations *(disposées enplaques)* were discovered, from which a corrosive ichor was discharged. A wash was employed, in which two drams of the medicine were dissolved, and afterwards an ointment composed of one third of hepar sulph. and two thirds of simple cerate.

2d. *Dartre squameuse* (scaly tetter).—A woman, three years since, was subject to eruptions sometimes on the back of the hands and feet, and sometimes on the face. These eruptions being principally on the face, their hideous appearance induced the patient to apply for advice. They appeared on the cheeks under the form of thick scales, at first white, then of an ash colour, going successively through all their periods to a state of desiccation, when they fell off to re-appear. Two ounces of hepar sulph. were dissolved in a bath for the whole body. The ointment was used as before, though somewhat weaker.—It is worth remarking, that the artificial baths of *Pere Elisée,* which he called *eau de Berrages,* were composed in this manner.

---

M. de Bierkin had under his care a girl twelve years of age, whose tongue was so large that its point fell upon the chin, causing the eversion of the lower lip, and giving to the anterior teeth an horizontal direction. The deglutition had become difficult, the voice unintelligible, and the larynx projected. The disease had existed from the age of two years, but once was cured by replacing it (as we suppose, by confining the tongue to its situation for a time). It was at length determined on extirpation, which was done with complete success. The tongue was pierced by a needle with double ligature in two places; the space between the ligatures was first tied, and then the lateral portions; a part of the tongue was suffered to remain. She recovered her voice and deglutition; the lip acquired its primitive situation; and the teeth, after being extracted, were replaced in a vertical direction, and properly secured.—*New-England Journal.*

---

Mr. WILLIAM SALISBURY, of the Botanic Garden, Sloane-street, at the recommendation of Drs. Babington and Curry, Richard Ogle, Esq. and other gentlemen, has lately adopted a mode of teaching Botany by making excursions into the fields near London, as was used to be practised by his late partner, Mr. William Curtis. It having become indispensably necessary for students in medicine to obtain a knowledge of this science, or at least so much of it as relates to an acquaintance with all the plants described in the materia medica, he is desirous of forming a regular establishment in the garden, for the purpose of rendering botanical information.—The Herborising Excursions will be made in certain days in every month, by meeting at Hampstead, Battersea Fields, and other places, noted for the production of different plants; and on these occasions, specimens will be gathered and named, their botanical as well as their medicinal qualities examined, and instructions given for forming them into a Hortus Siccus.—Mr. S. has appropriated a spot of ground in which are planted all the different vegetables used in medicine. A library of useful books in elucidation of the subject is likewise established in the garden; and those articles may be procured fresh as may be wanted in cases of emergency.—He also intends to set apart certain days during the season, when practical Lectures, more particularly explanatory of the classification of plants, phytology, &c. will be given in the library at the garden.

The above institution must be particularly useful in the present season, as, under the new Act, the examination in the materia medica is intended to be scrupulously strict as to the vegetable department; and, although young men may at present be excused from the neglect of this science, the public has a right to expect they will make themselves acquainted with all plants used in diet or medicine.

---

The Medical Lectures in the University of Glasgow will begin on Wednesday, November the 1st, at the following hours:—Institutions of Medicine, by Dr. Freer, at half-past eight in the morning; Surgery, by Dr. Jeffray, at ten; Midwifery, by Mr. Towers, at eleven; Practice of Medicine, by Dr. Freer, at twelve; Anatomy, by Dr. Jeffray, at two in the afternoon; Dietetics, Materia Medica, and Pharmacy, by Dr. Millar, at three; Chemistry and Chemical Pharmacy, by Dr. Cleghorn, at seven.—Dr. Brown will commence his Lectures on Botany about the beginning of May next.

The following gentlemen have obtained the Degree of Doctor in Medicine, from this University, within the last twelve months:—Mr. Hugh Bone, from Scotland; Mr. William Ferguson, from ditto; Mr. William Young, from ditto; Mr. William Macnish, from from ditto; Mr. Robert Hays, from Ireland; Mr. Benjamin M'Nair, from Scotland; Mr. James Arthur, from ditto.

*Medical Theatre, St. Bartholomew's Hospital.*—The following Courses of Lectures will be delivered at this theatre in the ensuing winter:—On the Theory and Practice of Medicine, by Dr. Hue. On Anatomy and Physiology, by Mr. Abernethy. On the Theory and Practice of Surgery, by Mr. Abernethy. On Chemistry, by Dr. Hue. On Midwifery, by Dr. Gooch. The Anatomical Demonstrations by Mr. Stanley.

The Anatomical Lectures will commence on Monday, October 2d, at two o'clock. Further particulars may be obtained by application to Mr. Wheeler, Apothecary, at the Hospital.

*Medical School of St. Thomas's and Guy's Hospitals.*—The Autumnal Course of Lectures at these adjoining Hospitals will commence in the beginning of October, viz.

At St. Thomas's—Anatomy and the Operations of Surgery, by Mr. Astley Cooper and Mr. Henry Cline. Principles and Practice of Surgery, by Mr. Astley Cooper.

At Guy's—Practice of Medicine, by Dr. Babington and Dr. Curry. Chemistry, by Dr. Babington, Dr. Marcet, and Mr. Allen. Experimental Philosophy, by Mr. Allen. Theory of Medicine, and Materia Medica, by Dr. Curry and Dr. Cholmeley. Midwifery, and Diseases of Women and Children, by Dr. Haighton. Physiology, or Laws of the Animal Œconomy, by Dr. Haighton. Structure and Diseases of the Teeth, by Mr. Fox.

*Medical, Chirurgical, and Chemical Lectures, at No. 9, George-street, Hanover-square, and No. 42, Great Windmill-street.*

1. The first Monday in October next, at No. 9, George-street, Hanover-square, a Course of Lectures on the Practice of Physic, with the Laws of the Animal Economy, will commence at nine in the morning, and be continued every succeeding Monday, Wednesday, and Friday, till February next, by George Pearson, M.D. F.R.S. Senior Physician to St. George's Hospital, of the College of Physicians, &c. &c.

2. On Tuesday, October 3d, at eight in the morning, on Chemistry, at No. 42, Windmill-street, to be continued every succeeding Tuesday, Thursday, and Saturday, at the same hour, by Augustus Granville, M.D.

3. On Tuesday, October 3d, at nine in the morning, on Therapeutics, with Materia Medica and Medical Jurisprudence, to be continued every succeeding Tuesday, Thursday, and Saturday, at the same hour, in Windmill-street, by Richard Harrison, M.D. Fellow of the College of Physicians, and Physician to the Northern Dispensary.

4. On Monday, October 2d, on the Theory and Practice of Surgery, at seven in the evening, to be continued every succeeding Monday, Wednesday, and Friday, at the same hour, in Windmill-street, by B. C. Brodie, F.R.S. Assistant-surgeon to St. George's Hospital.

*Theatre of Anatomy, Bartlett's-court, Holborn.*—Mr. John Taunton, F.A.S. Member of the Royal College of Surgeons of London, Surgeon to the City and Finsbury Dispensaries, City of London Truss Society, &c. will commence his Course of Lectures on Anatomy, Physiology, Pathology, and Surgery, on Saturday, October 7th, at eight o'clock in the evening precisely, and be continued every Tuesday, Thursday, and Saturday, at the same hour.

*Obstetrical Theatre*, 68, *Berner's-street.*—The winter Course of Lectures on the Science and Practice of Midwifery, including the Diseases of Women and Infants, by Dr. Clough, Physician-Accoucheur to the St. Mary-le-bone General Dispensary, &c. &c. will commence at his Theatre, on Monday morning the 2d of October, at half-past ten, and his evening Course at seven.

Dr. Adams will commence his autumnal Course of Lectures on the Institutes and Practice of Medicine, at ten o'clock in the morning, on the first Tuesday in October, at his house, No. 17, Hatton Garden. In these lectures all Mr. Hunter's doctrines will be perspicuously explained; and, under the division of Morbid Poisons, every variety of cutaneous diseases will be minutely attended to.

Dr. Ramsbotham will begin a Course of Lectures on Midwifery, on Wednesday, October 4th, at eleven in the morning, at the Theatre of the London Hospital.

In addition to the Anatomical Lectures and Demonstrations delivered in the Theatre of Anatomy, Great Windmill-street, Mr. Charles Bell will resume his Lectures on Surgery. The first division of the course will embrace the principles of surgery. The doctrine of wounds, and the duties of the army and navy surgeon, will form the second; and the third will consist of the operations of surgery performed on the dead body.

Dr. Clarke and Mr. Clarke will commence their Lectures on Midwifery, and the Diseases of Women and Children, on Wednesday, October 5th. The lectures are read at Mr. Clarke's house, 10, Saville-row, Burlington Gardens, every morning, from a quarter past ten to a quarter past eleven, for the convenience of students attending the hospitals.

Dr. Squire will, on Tuesday the 3d of October, begin a Course of Lectures on the Principles and Practice of Midwifery, and the Diseases of Women and Children.

Dr. Clutterbuck will begin his autumn Course of Lectures on the Theory and Practice of Physic, Materia Medica, and Chemistry, on Wednesday, October 4th, at ten o'clock in the morning, at his house, No. 1, in the Crescent, New Bridge-street, Blackfriars.

---

Mr. S. Rootsey has in the press, a Bristol Dispensary, which has for its object, 1st, to establish the nomenclature of pharmacy upon a permanent basis; and 2dly, to explain the advantages of a new method of expressing the composition of medicines.

London

### LONDON PRICES OF DRUGS.—August 25, 1815.

| | £. | s. | d. | £. | s. | d. per |
|---|---|---|---|---|---|---|
| Aloes Barbadoes, from | 22 | 0 | 0 to | 0 | 0 | 0 C. |
| ——Cape | 6 | 10 | 0.. | 7 | 0 | 0 — |
| ——Succotrina | 23 | 0 | 0.. | 0 | 0 | 0 — |
| ——Epatica or E.I. | 14 | 0 | 0.. | 16 | 0 | 0 — |
| Angelica Root | 8 | 0 | 0.. | 8 | 10 | 0 — |
| Alkanet Root | 8 | 10 | 0.. | 0 | 0 | 0 — |
| Antimony Crude | 2 | 18 | 0.. | 3 | 5 | 0 — |
| Aqua Fortis | S. | 0 | 8..D. | 1 | 1 | lb. |
| Arrow Root, fine | 0 | 2 | 0.. | 0 | 2 | 9 — |
| ——ordinary | 0 | 0 | 9.. | 0 | 1 | 3 — |
| Arsenic, Red | 4 | 4 | 0.. | 4 | 10 | 0 C. |
| ——White | 2 | 16 | 0.. | 0 | 0 | 0 — |
| Balsam Capivi | 0 | 4 | 10.. | 0 | 5 | 0 lb. |
| ——Peru | 1 | 0 | 0.. | 1 | 1 | 0 — |
| ——Tolu | 0 | 13 | 6.. | 0 | 14 | 0 — |
| Bark Jesuits', Com. | 0 | 3 | 6.. | 0 | 4 | 0 — |
| ——Second | 0 | 6 | 0.. | 0 | 7 | 0 — |
| ——Quil or best | 0 | 8 | 0.. | 0 | 10 | 0 — |
| ——Red | 0 | 8 | 8.. | 0 | 9 | 0 — |
| ——Yellow | 0 | 2 | 0.. | 0 | 2 | 2 — |
| Borax, refined E.I. | none. | | | | | |
| ——English | 0 | 2 | 8.. | 0 | 3 | 0 lb. |
| ——unrefined or Tinc. | 6 | 10 | 0.. | 7 | 15 | 0 C. |
| Camphire, refined | 0 | 6 | 0.. | 0 | 6 | 3 lb. |
| ——unrefined | 16 | 0 | 0.. | 17 | 10 | 0 C. |
| Cantharides | 0 | 9 | 6.. | 10 | 0 | 0 lb. |
| Cardamoms (best) | 0 | 6 | 6.. | 0 | 7 | 6 — |
| Cassia Buds | 32 | 0 | 0 } | 0 | 0 | 0 C. |
| ——Fistula, W.I. | 5 | 5 | 0 } | in bond | | — |
| ——Lignea | 42 | 0 | to } | 45 | 0 | 0 — |
| Castoreum American | 2 | 10 | 0.. | 3 | 0 | 0 lb. |
| ——Russia | 11 | 11 | 0.. | 0 | 0 | 0 — |
| Castor Oil, per bottle 1½ lb. | 0 | 3 | 9.. | 0 | 4 | 0 bo |
| Cocculus Indicus | 6 | 0 | 0.. | 7 | 0 | 0 C. |
| Colocynth Turkey | none. | | | | | |
| Columbo Root | 4 | 15 | 0.. | 6 | 0 | 0 C. |
| Cream of Tartar | 5 | 10 | 0.. | 6 | 0 | 0 — |
| Gallangal East-India | uncertain | | .. | 0 | 0 | 0 C. |
| Gentian Root | 3 | 10 | 0.. | 3 | 15 | 0 — |
| Ginseng | none | | .. | 0 | 0 | 0 lb. |
| Grains of Guinea | 6 | 10 | 0.. | 0 | 0 | 0 C. |
| Gum Ammo. Drop | 50 | 0 | 0.. | 0 | 0 | 0 — |
| ——Lump | 5 | 0 | 0.. | 7 | 0 | 0 — |
| ——Animi | 3 | 10 | 0.. | 7 | 10 | 0 — |
| ——Arabic, E.I. | 2 | 0 | 0.. | 4 | 0 | 0 — |
| ——Turkey, Fine | 9 | 0 | 0.. | 9 | 10 | 0 — |
| ——Barbary | 5 | 5 | 0.. | 0 | 0 | 0 — |
| ——Assafœtida | 10 | 0 | 0.. | 15 | 0 | 0 — |
| ——Benjamin | 15 | 0 | 0.. | 60 | 0 | 0 — |
| ——Gambogium | 28 | 0 | 0.. | 30 | 0 | 0 — |
| ——Copal, scraped | 0 | 5 | 9.. | 0 | 6 | 0 lb. |
| ——Galbanum | 28 | 0 | 0.. | 0 | 0 | 0 C. |

| | £. | s. | d. | £. | s. | d. per |
|---|---|---|---|---|---|---|
| Gum Guaiacum, from | 0 | 2 | 6 to | 0 | 3 | 9 lb. |
| ——Mastic | 0 | 4 | 6.. | 0 | 4 | 9 — |
| ——Myrrh | 15 | 0 | 0.. | 20 | 0 | 0 C. |
| ——Olibanum | 8 | 0 | 0.. | 11 | 0 | 0 — |
| ——Oppoponax | 80 | 0 | 0.. | 0 | 0 | 0 — |
| ——Sandrac | 8 | 0 | 0.. | 8 | 5 | 0 — |
| ——Senegal | 6 | 15 | 0.. | 7 | 0 | 0 — |
| ——Tragacanth | 26 | 15 | 0.. | 27 | 0 | 0 — |
| Jalap | 0 | 5 | 6.. | 0 | 5 | 9 lb. |
| Ipecacuanha | 0 | 14 | 6.. | 0 | 15 | 0 — |
| Isinglass Book | 0 | 9 | 0.. | 0 | 10 | 0 — |
| ——Leaf | 0 | 7 | 10.. | 0 | 8 | 6 — |
| ——Long Staple | 0 | 9 | 0.. | 0 | 10 | 6 — |
| ——Short Staple | 0 | 8 | 0.. | 0 | 8 | 6 — |
| Manna Flakey | 0 | 5 | 10.. | 0 | 6 | 6 — |
| ——Sicily in Sorts | 0 | 0 | 0.. | 0 | 0 | 0 — |
| Musk China | 0 | 14 | 0.. | 0 | 16 | 0 oz. |
| Nux Vomica | 2 | 0 | 0.. | 2 | 2 | 0 C. |
| Oil of Vitriol | 0 | 0 | 3¾ | 0 | 0 | 4 lb. |
| Opium, East-India | none | | .. | 0 | 0 | 0 — |
| ——Turkey | 1 | 4 | 6.. | 0 | 0 | 0 — |
| Pink Root | 0 | 4 | 0.. | 0 | 4 | 6 — |
| Quicksilver | 0 | 5 | 10.. | 0 | 0 | 0 — |
| Rhubarb, East-India | 0 | 9 | 0.. | 0 | 13 | 0 — |
| ——Russia | 0 | 18 | 0.. | 1 | 0 | 0 — |
| Saffron, Spanish | 2 | 16 | 0.. | 3 | 0 | 0 — |
| ——French | 1 | 18 | 0.. | 2 | 0 | 0 — |
| Sago | 6 | 15 | 0.. | 8 | 10 | 0 C. |
| Sal. Ammoniac | 10 | 10 | 0.. | 11 | 0 | 0 — |
| Sarsaparilla | 0 | 2 | 3.. | 0 | 4 | 9 lb |
| Sassafras | 3 | 0 | 0.. | 3 | 10 | 0 T. |
| Scammony, Aleppo | 1 | 14 | 0.. | 1 | 15 | 0 lb. |
| ——Smyrna | 0 | 18 | 0.. | 0 | 0 | 0 — |
| Senna, Alexandria | 0 | 4 | 0.. | 0 | 4 | 3 — |
| Seeds, Anni Alicant | 4 | 15 | 0.. | 5 | 0 | 0 C. |
| ——Coriander English | 0 | 14 | 0.. | 0 | 0 | 0 — |
| ——Cummin | 5 | 1 | 0.. | 0 | 0 | 0 — |
| ——Fenugreek | 1 | 15 | 0.. | 0 | 0 | 0 — |
| Shellack | 7 | 10 | 0.. | 10 | 15 | 0 — |
| Sticklack | 3 | 10 | 0.. | 7 | 0 | 0 — |
| Snake Root | 0 | 15 | 6.. | 0 | 0 | 0 lb. |
| Soap, Castile or Spanish | 9 | 0 | 0.. | 10 | 0 | 0 C. |
| Spermaceti, refined | 0 | 2 | 2.. | 0 | 0 | 0 lb. |
| Tamarinds, West-India | 9 | 0 | 0.. | 10 | 0 | 0 C. |
| Tapioca, Lisbon | 0 | 0 | 9.. | 0 | 1 | 0 lb. |
| Turmeric, Bengal | 4 | 10 | 0.. | 4 | 15 | 0 C. |
| ——China | 6 | 10 | 0.. | 7 | 0 | 0 — |
| ——West-India | 3 | 10 | 0.. | 4 | 4 | 0 — |
| Verdigris, Wet | 0 | 3 | 6.. | 0 | 0 | 0 lb. |
| ——Dry | 0 | 6 | 6.. | 0 | 0 | 0 — |
| ——Crystallized | 0 | 8 | 0.. | 0 | 0 | 0 — |
| Vitriol, Roman | 0 | 0 | 7.. | 0 | 0 | 0 — |
| ——Foreign white | 2 | 10 | 0.. | 0 | 0 | 0 C. |

*Price of Vials per Gross.*—8 oz. 70s.—6 oz. 58s.—4 oz. 47s.—3 oz. 43s.—2 oz. 36s.—1 oz. 30s.—½ oz. 24s.

Lisbon Leeches, 30s. per hundred.—True French ditto, 55s.

Essential Salt of Lemons, 4s. 6d. per dozen.

### METEOROLOGICAL REGISTER.

*From July the 25th, to August the 25th,* 1815.

Kept by C. BLUNT, Philosophical Instrument Maker, No. 38, Tavistock-Street, Covent-Garden.

| Moon. | Day. | Wind. | Barometrical Pressure. Max. | Min. | Mean. | Temperature. Max. | Min. | Mean. | |
|---|---|---|---|---|---|---|---|---|---|
| | 26 | N | 30·10 | 30·06 | 30·085 | 68 | 46 | 57 | Fair |
| | 27 | NE | 30·13 | 30·09 | 30·11 | 69 | 48 | 58·5 | Fair |
| | 28 | NW | 30·13 | 30·10 | 30·12 | 72 | 49 | 60·5 | Fair |
| ◑ | 29 | W | 30·10 | 30·02 | 30·04 | 74 | 53 | 63·5 | Fair |
| | 30 | E by S | 30·05 | 30·05 | 30·05 | 78 | 52 | 65 | Fair |
| | 31 | E | 30·13 | 30·06 | 30·09 | 75 | 46 | 60·5 | Fair |
| | 1 | E | 30·19 | 30·16 | 30·182 | 74 | 53 | 63·5 | Fair |
| | 2 | NW | 30·13 | 30·06 | 30·107 | 76 | 52 | 64 | Fair, a slight shower p. m. |
| | 3 | NW | 30·21 | 30·13 | 30·182 | 77 | 51 | 64 | Fair |
| | 4 | W | 30·09 | 29·95 | 30·015 | 75 | 50 | 62·5 | Fair |
| ● | 5 | W | 29·78 | 29·66 | 29·712 | 70 | 48 | 59 | Cloudy |
| | 6 | NE | 29·80 | 29·80 | 29·80 | 66 | 48 | 57 | Rain |
| | 7 | NE | 29·96 | 29·94 | 29·83 | 70 | 45 | 57·5 | Rain |
| | 8 | N | 29·94 | 29·92 | 29·925 | 71 | 49 | 60 | Fair |
| | 9 | NW | 29·97 | 29·94 | 29·962 | 71 | 48 | 59·5 | Fair |
| | 10 | N | 29·92 | 29·81 | 29·86 | 73 | 53 | 63 | Fair |
| ◐ | 11 | NW | 29·56 | 29·53 | 29·545 | 70 | 44 | 57 | Rain during the Night |
| | 12 | N | 29·69 | 29·60 | 29·647 | 71 | 48 | 59·5 | Rain at 8 p.m. |
| | 13 | N | 29·90 | 29·90 | 29·90 | 72 | 49 | 60·5 | Fair |
| | 14 | W by N | 30·09 | 30·07 | 30·08 | 72 | 48 | 60 | Fair |
| | 15 | W | 30·10 | 30·04 | 30·065 | 74 | 57 | 65·5 | Fair |
| | 16 | W | 29·80 | 29·67 | 29·735 | 73 | 50 | 61·5 | Fair, Rain during Night |
| | 17 | W | 29·99 | 29·94 | 29·972 | 76 | 49 | 62·5 | Fair |
| | 18 | W | 29·97 | 29·80 | 29·885 | 75 | 50 | 62·5 | Fair |
| ○ | 19 | W | 29·83 | 29·75 | 29·785 | 74 | 45 | 59·5 | Rain |
| | 20 | NW | 29·90 | 29·90 | 29·90 | 70 | 49 | 59·5 | Fair |
| | 21 | N | 30·02 | 29·97 | 29·99 | 71 | 50 | 60·5 | Fair |
| | 22 | W | 29·83 | 29·64 | 29·70 | 73 | 57 | 65 | Frequent heavy showers |
| | 23 | W | 29·77 | 29·66 | 29·697 | 72 | 57 | 64·5 | Frequent heavy showers |
| | 24 | SW | 30·08 | 29·98 | 30·03 | 73 | 57 | 65 | Fair |
| | 25 | S | 30·08 | 30 | 30·046 | 80 | 57 | 68·5 | Fair |

RESULTS.

Mean barometrical pressure of the month 29·946
Maximum 30·19, wind at E
Minimum 29·53, ——— NW

Mean temperature of the month 61·3 deg.
Maximum 80, wind at S
Minimum 44, ——— NW

*Scale exhibiting the prevailing Winds during the Month.*

| N | NE | E | SE | S | SW | W | NW |
|---|---|---|---|---|---|---|---|
| 6 | 3 | 2 | 1 | 1 | 1 | 10 | 7 |

| | Mean barometrical pressure. | Mean temperature. |
|---|---|---|
| From the last quarter on the 29th July, to the new moon on the 5th of August | 30·095 | 63·2 |
| ——— new moon on the 5th, to the first quarter on the 11th | 29·484 | 59·3 |
| ——— first quarter on the 11th, to the new moon on the 19th | 29·853 | 61·1 |

REPORT

### REPORT OF DISEASES.

The general character of the diseases still continues inflammatory, notwithstanding the autumnal period is so far advanced. Even the diarrhœas which have been frequent are attended with more pain than usual; and where dysentery has supervened, the tormina have been so considerable as to shew evident marks of high action. Bleeding has relieved them in all the cases in which it has been tried.

Scarlatina has been very general, and not less mild, confirming the sagacious remark of Dr. Willan concerning the effect of seasons in this contagion. The terror of it, however, has evacuated the principal public school in the north side of the metropolis, but we have not heard of any fatal cases. The throat has, in most instances which came under the Reporter's observation, shewn great inflammation, but the sloughs separated kindly, leaving, in some cases, salivation; in others, the sequelæ have been as usual anasarca or rheumatism. Either has yielded to brisk purges.

A singular case of fits has occurred in a boy about fourteen years old. In many respects they appeared epileptic, but the senses and memory were not impaired. On a sudden all the marks of chorœa appeared in the right hand, and afterwards in the leg of the same side. At the same time the pain in the head was so alarming as to indicate cupping or leeches. When much reduced by these, he was assisted by chalybeates; but the head-ach returned, and was much relieved by epistaxis. This case promises to be interesting, as partaking of those anomalous symptoms which so often puzzle the doctor at that interesting and important period of life.

MONTHLY

